# Supplementary material for: Dengue Severity Prediction in a Hyperendemic Region in Colombia
Source: Viruses. 2025 May 22;17(6):740. doi: 10.3390/v17060740 (PMC12197464; doi:10.3390/v17060740)
Supplement: Supplementary file 1 [file viruses-17-00740-s001.zip › Supplementary Material S1. STROBE Statement-checklist.pdf]

Dengue severity prediction in a hyperendemic region in Colombia

Supplementary Material S1. STROBE Statement—checklist of items that should be included in reports of observational studies

	Item No.	Recommendation	Page No.	Relevant text from manuscript
**Title and abstract**	1	(*a*) Indicate the study's design with a commonly used term in the title or the abstract	3	"A cross-sectional analysis was conducted using data from 2018 to 2022, encompassing 233 patients […]"
		(*b*) Provide in the abstract an informative and balanced summary of what was done and what was found	3	"Associations between clinical, demographic, and laboratory data and disease severity were examined using Fisher's exact tests or the Mann-Whitney U test (p<0.05). Profiles for SD and non-SD cases were established through multiple correspondence analysis, and a logistic regression-based predictive model was validated using training and test sets. The model's performance was evaluated using the Area Under the Receiver Operating Characteristic Curve (AUC-ROC), accuracy, sensitivity, F1-score, and precision. […]Differences in place of residence, comorbidities, type of infection, and signs and symptoms were observed between the severe dengue (SD) and non-severe dengue (non-SD) groups. Median levels of platelets, white blood cells (WBC), aspartate aminotransferase (AST), and Alanine Aminotransferase (ALT) were found to be higher in the SD group compared to the non-SD group. Neutrophils, leukocytes,

				platelets, AST, and primary infection were significant predictors of SD. The model demonstrated an area under the receiver operating characteristic curve (AUC) of 0.91 (95% CI, 0.85-0.96)"
		**Introduction**		
Background/rationale	2	Explain the scientific background and rationale for the investigation being reported	5-7	
Objectives	3	State specific objectives, including any prespecified hypotheses	7	"[…] this study seeks to develop a predictive model for severe dengue, using data from Urabá, a hyperendemic region in Colombia."
		**Methods**		
Study design	4	Present key elements of study design early in the paper	8	"A descriptive cross-sectional observational study was conducted […]"
Setting	5	Describe the setting, locations, and relevant dates, including periods of recruitment, exposure, follow-up, and data collection	8	"A descriptive cross-sectional observational study was conducted, adhering to the STROBE checklist guidelines, detailed in Supplementary Material 1. Figure 1 depicts the flow of the sample used for predictive analysis. This study analyzed data from 2018 to 2022, including 233 patients. Data were sourced primarily from two cohorts. The first source was a prospective cohort of newly identified cases from November 2020 to September 2022, which included 192 patients, 6 of whom had severe cases. Owing to the small number of severe cases in this cohort, a second data source was added, featuring 41 retrospective cases from two distinct periods: 2018 to 2019 and October to December 2022. Among the 47 patients diagnosed with severe

				dengue, symptoms included severe hemorrhage (n=6), significant organ damage (n=17), and extensive fluid leakage (n=24)."
Participants	6	(*a*) *Cohort study*—Give the eligibility criteria, and the sources and methods of selection of participants. Describe methods of follow-up<br>*Case-control study*—Give the eligibility criteria, and the sources and methods of case ascertainment and control selection. Give the rationale for the choice of cases and controls<br>*Cross-sectional study*—Give the eligibility criteria, and the sources and methods of selection of participants	8-9	"Patients were recruited through active searches in both hospital databases and community settings. Trained nurses carried out epidemiological surveillance during outbreaks, visiting homes to connect with patients who had sought medical help. The cohort included individuals of all ages, genders, and ethnicities presenting with fever lasting up to 7 days, without a clear infectious source, and showing at least two of the following symptoms: headache, pain behind the eyes, muscle pain, joint pain, or skin rash. Confirmation of dengue was determined through specific laboratory tests, ensuring all participants met the critical diagnostic criteria. Exclusion criteria included individuals who had received blood product transfusions within three months before the study or those with a history of blood disorders. Patients were assessed during their febrile phase and followed through the recovery phase, until day 14 or 21, marking the resolution of symptoms.<br>Retrospective cases were identified by reviewing clinical records. Distribution of these cases included 11 from 2022, 4 from 2020, 3 from 2019, 22 from 2018, and 1 from 2016. It is important to note that these additional cases lacked the detailed information gathered for

				the original cohort, as they were not subjected to all the initial analyses.”
		(*b*) *Cohort study*—For matched studies, give matching criteria and number of exposed and unexposed<br>*Case-control study*—For matched studies, give matching criteria and the number of controls per case	Not applicate	
Variables	7	Clearly define all outcomes, exposures, predictors, potential confounders, and effect modifiers. Give diagnostic criteria, if applicable	10	“Cases were categorized according to the WHO's 2009 dengue guidelines [5] into two groups: non-severe dengue (non-SD) and severe dengue (SD). A case was classified as SD if it met any of the following criteria: a) Evidence of shock or accumulation of pleural/peritoneal fluid leading to respiratory failure, b) Severe bleeding requiring medical intervention, c) Significant impairment of vital organs, such as acute liver failure (defined as AST and ALT levels ≥ 1000 U/L), acute kidney injury (AKI) with a serum creatinine level increase of ≥ 0.3 mg/dL within 48 hours or an elevation of ≥ 1.5 times from baseline within 7 days, encephalopathy (evidenced by seizures or disturbances in consciousness), or signs of myocarditis or heart failure”.
Data sources/ measurement	8*	For each variable of interest, give sources of data and details of methods of assessment (measurement). Describe comparability of assessment methods if there is more than one group	9	“Eligibility criteria for study participants required laboratory-confirmed dengue virus (DENV) infection, with the satisfaction of criteria of one of the following: 1) Positive DENV-specific real-time reverse transcription polymerase chain reaction (RT-PCR); this test

				was conducted on all serum samples collected during the acute phase, following RNA extraction, 2) Confirmation of the dengue virus NS1 antigen using capture ELISA on acute-phase serum samples, or 3) seroconversion IgM or IgG antibodies [5]. The identification of viral RNA through RT-PCR was conducted utilizing a CDC dengue virus serotype detection kit, in adherence to the manufacturer's guidelines. IgM antibody detection was assessed using the Panbio capture ELISA method, following the manufacturer's protocol, across both acute and convalescent phase serum samples. IgG antibody detection was carried out using the Focus capture ELISA method, following the manufacturer's instructions. This process was applied to all acute phase serum samples, with repeat testing during the convalescent phase 14 days later in cases of initial negative outcomes […]"
Bias	9	Describe any efforts to address potential sources of bias	25-26	This study is not without its limitations. The challenges we faced were closely tied to the broader healthcare context of the period studied. While commitment to using complete data records strengthened the authenticity and reliability of the findings, it also limited the sample size, potentially affecting the generalizability of our conclusions. Furthermore, the complex clinical manifestations of SD, particularly hemodynamic shifts, may interact in intricate

			ways with various prognostic factors, adding complexity to disease progression assessments. Another aspect that significantly impacted the study is the reliance on secondary data (41 cases of severe dengue), which has implications such as the lack of data on key variables like serotypes, types of infection, and liver function. In some instances, the original variables were retained, while in others, where significant data loss occurred, imputation was employed. Although this was done in a controlled manner, the absence of data may affect the findings.<br>The temporal scope of study, especially from 2020 onwards, intersected with the COVID-19 pandemic, presenting unique challenges. For instance, the data from 2020 to 2022 recorded 213 dengue cases, but this number may not accurately reflect the true prevalence due to pandemic-related disruptions. Symptomatic similarities between dengue and COVID-19, along with the heightened focus on the latter, could have led to underreporting of dengue cases. Misdiagnoses due to COVID-19 concerns and patients' reluctance to seek medical care due to fear of COVID-19 exposure might have resulted in underreported dengue cases. The inclusion of data from 2018 and 2019 helped mitigate this issue to some extent, but the impact of the pandemic on dataset cannot be ignored.”
Study size	10	Explain how the study size was arrived at	Not applicate

Quantitative variables	11	Explain how quantitative variables were handled in the analyses. If applicable, describe which groupings were chosen and why	11	"[…] Comparisons between the SD and non-SD groups were made using Fisher's tests for qualitative variables and Mann-Whitney U tests for quantitative variables. A p-value below 0.05 was considered statistically significant."
Statistical methods	12	(*a*) Describe all statistical methods, including those used to control for confounding	13-14	"Three machine learning models -Logistic Regression, Decision Tree Classifier, and Random Forest Classifier- were assessed through 10-fold cross-validation on the training data. The logistic regression model demonstrated a mean accuracy of 0.89 with a standard deviation of 0.08, outperforming the other models tested. The support vector machine reported a mean accuracy of 0.87 and a standard deviation of 0.06. The decision tree model had a mean accuracy of 0.82 with a standard deviation of 0.07, and the random forest model showed a mean accuracy of 0.89 but with a lower standard deviation of 0.04 compared to logistic regression. Despite the similar mean accuracy values between logistic regression and random forest, logistic regression was chosen for further optimization due to its higher interpretability, which is crucial for clinical applications. Hyperparameter tuning was performed via Grid Search, examining various parameters including

regularization strengths (C), penalty types (l1, l2, and elasticnet), the mix of l1 and l2 in elasticnet, and the optimization algorithm (saga). The optimal parameters were identified after conducting another round of 10-fold cross-validation. Logistic Regression is advantageous as it allows the use of regularization techniques to prevent overfitting, a common challenge in predictive modeling. In this study, the Grid Search method was employed to systematically explore a range of hyperparameters to find the optimal combination, thereby enhancing the model's accuracy and generalizability. Specifically, the term 'elasticnet' refers to a type of regularization used during this optimization process. Elasticnet is a hybrid approach that combines the strengths of L1 and L2 regularization methods. This blend is particularly beneficial for dealing with correlated predictors as it helps in balancing variable selection and model complexity, effectively preventing overfitting. These techniques together ensure the development of a robust model that not only reliably predicts outcomes but also maintains clarity and simplicity, preventing the model from becoming overly complex.

Prior to training, Z-score normalization was applied to ensure uniform variable contribution. The dataset was split into an 80% training subset and a 20% testing subset. Post-hyperparameter tuning, the model's performance was assessed on both subsets using metrics like accuracy, precision, recall, F1 score, ROC curve, and AUC with its 95% CI. AUC benchmarks were classified as follows: 0.90-1 (excellent), 0.80-0.90 (good), 0.70-0.80 (fair), 0.60-0.70 (below average), and 0.50-0.60 (inadequate) [25].

Before finalizing the Logistic Regression model's conclusions, foundational assumptions were validated. This included verifying the linear relationship between independent variables and log-odds, assessing multicollinearity using the variance inflation factor (VIF), identifying significant outliers and influential points using residual plots and Cook's distance, and evaluating model fit using the Hosmer-Lemeshow test. A non-significant result ($p > 0.05$) indicated an appropriate model fit. The model was built using variables that demonstrated a statistically significant in the bivariate analysis. The exclusion process considered the restrictions of the predictive model based on the number of events per predictor and clinical relevance. A key criterion for

			excluding variables from the final model was high collinearity between predictors (e.g., only one liver function test was retained from those available).<br>To assess the impact of individual predictors, Odds Ratios (ORs) were calculated using a generalized linear model (GLM) with a logit link function. ORs were derived directly from the coefficients of the predictors. To ensure the robustness of these estimates, a bootstrapping methodology with 1,000 repetitions was employed to generate 95% confidence intervals (CIs). All statistical analyses were conducted using Python 3.11.5 and R 4.3.1, which provide comprehensive computational toolkits for the statistical and machine learning evaluations integral to this study. This dual-software approach leverages the strengths of both environments, enhancing the analytical capabilities necessary for rigorous data analysis.”.
	(*b*) Describe any methods used to examine subgroups and interactions	Not applicate	
	(*c*) Explain how missing data were addressed	11-12	“During the data analysis phase, we encountered inconsistencies in the form of missing data, especially in sections related to laboratory tests, hemograms, and liver function tests. To address these gaps, a rigorous data imputation technique was employed. Missing values were replaced

			with the average of the available data, calculated with precision. To enhance accuracy, this average was computed using data from individuals of the same gender and within a five-year age range of the patient with missing data. Notably, approximately 25% of the liver function test results required imputation, and less than 5% of hemoleucogram values had missing data that needed attention. This method ensured the accuracy and clinical relevance of the imputations, preserving the integrity and credibility of our dataset while minimizing potential biases.”
	(*d*) *Cohort study*—If applicable, explain how loss to follow-up was addressed<br>*Case-control study*—If applicable, explain how matching of cases and controls was addressed<br>*Cross-sectional study*—If applicable, describe analytical methods taking account of sampling strategy	Not applicate	
	(*e*) Describe any sensitivity analyses	13	The dataset was split into an 80% training subset and a 20% testing subset. Post-hyperparameter tuning, the model's performance was assessed on both subsets using metrics like accuracy, precision, recall, F1 score, ROC curve, and AUC with its 95% CI. AUC benchmarks were classified as follows: 0.90-1 (excellent), 0.80-0.90 (good), 0.70-0.80 (fair), 0.60-0.70 (below average), and 0.50-0.60 (inadequate) [25].

**Results**				
Participants	13*	(a) Report numbers of individuals at each stage of study—eg numbers potentially eligible, examined for eligibility, confirmed eligible, included in the study, completing follow-up, and analysed	14	"In the study, data from 233 patients, all confirmed with DENV infections, were analyzed. The median age was 10 years, with an interquartile range of 11 years. Of these, 47 progress to severe dengue (SD) cases (28 males and 19 females), and 186 as non-severe dengue (non-SD) cases (105 males and 81 females), according to the WHO 2009 criteria […] "
		(b) Give reasons for non-participation at each stage	Not applicate	
		(c) Consider use of a flow diagram	Not applicate	
Descriptive data	14*	(a) Give characteristics of study participants (eg demographic, clinical, social) and information on exposures and potential confounders	15-21	Table 1, Table 2, Table 3
		(b) Indicate number of participants with missing data for each variable of interest	15-21	Table 1, Table 2, Table 3
		(c) *Cohort study*—Summarise follow-up time (eg, average and total amount)	8	"Patients were recruited through active searches in both hospital databases and community settings. Trained nurses carried out epidemiological surveillance during outbreaks, visiting homes to connect with patients who had sought medical help. The cohort included individuals of all ages, genders, and ethnicities presenting with fever lasting up to 7 days, without a clear infectious source, and showing at least two of the following symptoms: headache, pain

				behind the eyes, muscle pain, joint pain, or skin rash. Confirmation of dengue was determined through specific laboratory tests, ensuring all participants met the critical diagnostic criteria. Exclusion criteria included individuals who had received blood product transfusions within three months before the study or those with a history of blood disorders. Patients were assessed during their febrile phase and followed through the recovery phase, until day 14 or 21, marking the resolution of symptoms.”
Outcome data	15*	*Cohort study*—Report numbers of outcome events or summary measures over time	15-21	Table 1, Table 2, Table 3, Table 4, Figure 2, Figure 3
		Case-control study—Report numbers in each exposure category, or summary measures of exposure	Not applicate	
		Cross-sectional study—Report numbers of outcome events or summary measures	15-21	Table 1, Table 2, Table 3, Table 4, Figure 2, Figure 3
Main results	16	(*a*) Give unadjusted estimates and, if applicable, confounder-adjusted estimates and their precision (eg, 95% confidence interval). Make clear which confounders were adjusted for and why they were included	15-21	Table 1, Table 2, Table 3, Table 4, Figure 2, Figure 3
		(*b*) Report category boundaries when continuous variables were categorized	Not applicate	
		(*c*) If relevant, consider translating estimates of relative risk into absolute risk for a meaningful time period	Not applicate	

Other analyses	17	Report other analyses done—eg analyses of subgroups and interactions, and sensitivity analyses	15-21	Table 4, Figure 2, Figure 3
**Discussion**				
Key results	18	Summarise key results with reference to study objectives	21	"This study revealed that patients with severe dengue (SD) frequently exhibited symptoms like irritability, fluid accumulation, abdominal pain, hepatomegaly, hematemesis, and enlargement of organs such as the spleen and liver. In contrast, patients with non-severe dengue often lacked these specific symptoms but were more likely to display a rash and other milder manifestation. Multivariable predictive model identified secondary infections, along with platelet, leukocyte, neutrophil, and AST levels, as significant predictors of SD."
Limitations	19	Discuss limitations of the study, taking into account sources of potential bias or imprecision. Discuss both direction and magnitude of any potential bias	25-26	"This study is not without its limitations. The challenges we faced were closely tied to the broader healthcare context of the period studied. While commitment to using complete data records strengthened the authenticity and reliability of the findings, it also limited the

sample size, potentially affecting the generalizability of our conclusions. Furthermore, the complex clinical manifestations of SD, particularly hemodynamic shifts, may interact in intricate ways with various prognostic factors, adding complexity to disease progression assessments. Another aspect that significantly impacted the study is the reliance on secondary data (41 cases of severe dengue), which has implications such as the lack of data on key variables like serotypes, types of infection, and liver function. In some instances, the original variables were retained, while in others, where significant data loss occurred, imputation was employed. Although this was done in a controlled manner, the absence of data may affect the findings.

The temporal scope of study, especially from 2020 onwards, intersected with the COVID-19 pandemic, presenting unique challenges. For instance, the

				data from 2020 to 2022 recorded 213 dengue cases, but this number may not accurately reflect the true prevalence due to pandemic-related disruptions. Symptomatic similarities between dengue and COVID-19, along with the heightened focus on the latter, could have led to underreporting of dengue cases. Misdiagnoses due to COVID-19 concerns and patients' reluctance to seek medical care due to fear of COVID-19 exposure might have resulted in underreported dengue cases. The inclusion of data from 2018 and 2019 helped mitigate this issue to some extent, but the impact of the pandemic on dataset cannot be ignored.”.
Interpretation	20	Give a cautious overall interpretation of results considering objectives, limitations, multiplicity of analyses, results from similar studies, and other relevant evidence	21-26	
Generalisability	21	Discuss the generalisability (external validity) of the study results	21-26	
**Other information**				
Funding	22	Give the source of funding and the role of the funders for the present study and, if applicable, for the original study on which the present article is based	2	“This research received funding from the Ministry of Science, Technology and

			Innovation -Minciencias-, Colombia (https://minciencias.gov.co/) [grand number 325684267882]. The funders had no role in study design, data collection and analysis, publication decisions, or manuscript preparation.”

*Give information separately for cases and controls in case-control studies and, if applicable, for exposed and unexposed groups in cohort and cross-sectional studies.

Note: An Explanation and Elaboration article discusses each checklist item and gives methodological background and published examples of transparent reporting. The STROBE checklist is best used in conjunction with this article (freely available on the Web sites of PLoS Medicine at http://www.plosmedicine.org/, Annals of Internal Medicine at http://www.annals.org/, and Epidemiology at http://www.epidem.com/). Information on the STROBE Initiative is available at www.strobe-statement.org.
