# Supplementary material for: Dengue Severity Prediction in a Hyperendemic Region in Colombia
Source: Viruses. 2025 May 22;17(6):740. doi: 10.3390/v17060740 (PMC12197464; doi:10.3390/v17060740)
Supplement: Supplementary file 1 [file viruses-17-00740-s001.zip › Supplementary Material S2. No significant variables.pdf]

Dengue severity prediction in a hyperendemic region in Colombia

Supplement Material S2.

Signs and symptoms stratified by Dengue Severity versus non-DS (No significant differences)

**Variable**	**SD**		**Non-SD**		**OR**	**p-value a**
	**n=47**	**%**	**n=186**	**%**		
Headache	35	74.5	160	86.0	0.47	0.080
Diarrhea	17	36.2	62	33.3	1.13	0.732
Asthenia	30	63.8	128	68.8	0.80	0.600
Chills	21	44.7	108	58.1	0.58	0.104
Facial edema	8	17.0	30	16.1	1.07	0.828
Adenopathies	3	6.4	3	1.6	4.16	0.100
Petechiae	11	23.4	51	27.4	0.81	0.712
Epistaxis	6	12.8	27	14.5	0.86	1.000
Gingival bleeding	9	19.1	16	8.6	2.52	0.060
Ecchymosis	6	12.8	13	7.0	1.95	0.231
Tourniquet test	2	4.3	40	21.5	1.04	1.000
Cough	5	10.6	31	16.7	0.60	0.373
Bleeding mucosa	6	12.8	36	19.4	0.61	0.400
Manes	8	17.0	24	12.9	1.38	0.480
Metrorrhagia	1	2.1	5	2.7	0.72	1.000
Diabetes	0	0.0	2	1.1	Na	1.000
Dyslipidemia	1	2.1	0	0.0	Na	0.456
Cardiovascular	0	0.0	5	2.7	Na	0.567
Kidney	2	4.3	1	0.5	8.20	0.195
Autoimmune	2	4.3	1	0.5	8.20	0.195
Liver	7	14.9	7	3.8	4.50	0.012
Neoplasia	1	2.2	0	0.0	Na	0.456
Hypothyroidism	1	2.1	0	0.0	Na	0.456
Anemia	1	2.1	0	0.0	Na	0.456
Obesity	1	2.1	0	0.0	Na	0.456

Note: Na: Not applicable. SD: Severe Dengue. OR: Odds Ratio. CI: confidence interval. a: test of Fisher
